# Supplementary material for: Younger Americans are less politically polarized than older Americans about climate policies (but not about other policy domains)
Source: PLoS One. 2024 May 15;19(5):e0302434. doi: 10.1371/journal.pone.0302434 (PMC11095675; doi:10.1371/journal.pone.0302434)
Supplement: S1 Appendix — (DOCX) [file pone.0302434.s001.docx]

**S1 Appendix. Complete Question Wording and Coding Decisions**

**Independent Variables**

**Liberal-Conservative Political Ideology**: ANES asked participants “Where would you place yourself on this scale, or haven’t you thought much about this?” 7-point scale; 1 = *extremely liberal*, 4 = *moderate; middle-of-the-road*, 7 = *extremely conservative*.

**Age**: Respondent age was computed by asking respondents to list their birth year, month and day, then age was calculated by subtracting the respondents’ self-report birthdate from the date the interview was conducted.

**Control variables** (used in all analyses)

**Education**: ANES reports a “summary” variable for educational attainment, describing the highest level of education completed by each respondent. This variable is built from smaller questions like “how many grades of primary school did you complete?” and “do you hold any educational degrees?” This variable was then transformed into a binary variable, with 1 representing people who had completed at least a college degree (including community college and two-year programs), and 0 for everyone else.

**Gender**: ANES asked respondents to self-report their sex, either “male” or “female”. This variable was analyzed as a factor variable, with levels “female” (set as the reference gender) and “male”.

**Household Income**: ANES assessed household income in 2020 via self-report, asking for pre-tax household income in the previous year. In most other years, however, respondents indicated their household income range using a list of income intervals (e.g. $5,000 – $10,000, over $125,000; see Supporting Information). This variable is modeled as continuous, and income ranges were coded into integer values by taking the lower end of the range.

**Climate policy time-series items**

**Federal Spending on the Environment** (four versions)

Version 1 [ANES 1982]: Is the government spending too much, too little, or about the right amount on improving and protecting the environment? This version of the question was coded as 0 = *too much* or *about the right amount* and 1 = *too little*. This was done to align direction of answers with other ANES waves.

Version 2 [ANES 1984, 1986, 1988, 1990, 1992, 1994, 1996]: Should federal spending on improving and protecting the environment be increased, decreased, or kept about the same?

Version 3 [ANES 2000, 2002]: Should federal spending on Environmental Protection be increased, decreased, or kept about the same?

Version 4 [ANES 2008, 2012, 2016, 2020]: Should federal spending on protecting the environment be increased, decreased, or kept the same?

In 2008 and 2020, a branching question format was used for this question to produce a 7-point scale response to this question (“should spending be [increased / decreased] by [a lot / a moderate amount / a little]?”). We dichotomize this variable (for 2020 and 2008) for the time-series analysis to standardize response formats across ANES survey waves.

**Environmental regulations versus business interests trade-off** (three versions)

Version 1 [ANES 1996, 1998, 2000, 2020]: Some people think we need much tougher government regulations on business in order to protect the environment. Suppose these people are at one end of a scale, at point 1. Others think that current regulations to protect the environment are already too much of a burden on business. Suppose these people are at the other end, at point 7. And, of course, some other people have opinions somewhere in between, at points 2,3,4,5, or 6. Where would you place yourself on this scale, or haven’t you thought much about this?

Version 2 [ANES 1996, 2000, 2004, 2008]: Some people think it is important to protect the environment even if it costs some jobs or otherwise reduces our standard of living. (Suppose these people are at one end of the scale, at point number 1) Other people think that protecting the environment is not as important as maintaining jobs and our standard of living. (Suppose these people are at the other end of the scale, at point number 7. And of course, some other people have opinions somewhere in between, at points 2, 3, 4, 5, or 6). Where would you place YOURSELF on this scale, or haven't you thought much about this?

Version 3 [ANES 2012, 2016]: Some people think the federal government needs to regulate business to protect the environment. They think that efforts to protect the environment will also create jobs. Let us say this is point 1 on a 1-7 scale. Others think that the federal government should not regulate business to protect the environment. They think this regulation will not do much to help the environment and will cost us jobs. Let us say this is point 7 on a 1-7 scale. And of course, some other people have opinions somewhere in between, at points 2,3,4,5, or 6. Where would you place yourself on this scale, or haven't you thought much about this?

Responses to all versions of this question were reverse-scored, such that 1 indicated maximal preference for business interests, and 7 represented maximal preference for greater environmental regulations.

ANES used three different question wordings for this item over different survey waves, but always assessed whether to prioritize environmental regulations versus business interests. In ANES 1996, 1998, 2000 and 2020, the question framed the policy trade-off as environmental regulations versus the burden they place on businesses. In ANES 1996, 2000, 2004 and 2008, the question pitted environmental regulations versus “maintaining jobs and standard of living”. In ANES 2012 and 2016, the question framed positive aspects of environmental regulations (protecting the environment and the jobs such regulations would create) against negative aspects of environmental regulations (“regulations would not do much to help the environment and will cost jobs”).

We averaged responses to the two different versions of this question in 1996 and 2000 in the time-series analysis, based on high item correlations (Pearson’s *r* = 0.54 in 1996, Pearson’s *r* = 0.45 in 2000).

In 2000, ANES asked two versions each of Environment vs Jobs and Environment vs Business; one version for standard face-to-face interviewing using 7-point scales, and another for telephone interviewing using branching question format to produce a 5-point scale. ANES produced summary variables for both Environment vs Jobs and Environment vs Business that collapse across the two interviewing formats into a unified 5-point scale; these is the variable we analyze in the manuscript. After standardizing model outputs, regression coefficients for the 5-point questions become comparable to all other years using 7-point versions of the questions.

**Non-climate time-series items**

**Defense Spending** (one version)

[ANES 1982, 1984, 1986, 1988, 1990, 1992, 1994, 1996, 2000, 2004, 2008, 2012, 2016, 2020]: Some people believe that we should spend much less money for defense. (Suppose these people are at one end of a scale, at point 1.) Others feel that defense spending should be greatly increased. (Suppose these people are at the other end, at point 7.) And, of course, some other people have opinions somewhere in between, at points 2, 3, 4, 5 or 6. Where would you place yourself on this scale, or haven’t you thought much about this?

This question was reverse scored, such that 1 reflected the typical political conservatives’ position (preference for greatly *increasing* federal spending on defense), while 7 represented the typical liberal viewpoint (preference for greatly *decreasing* federal spending on defense).

**Private vs Public Health Insurance** (one version)

[ANES 1984, 1988, 1992, 1996, 2000, 2004, 2008, 2012, 2016, 2020]: There is much concern about the rapid rise in medical and hospital costs. Some people feel there should be a government insurance plan which would cover all medical and hospital expenses for everyone. Suppose these people are at one end of a scale, at point 1. Others feel that all medical expenses should be paid by individuals through private insurance plans like Blue Cross or other company paid plans. Suppose these people are at the other end, at point 7. And, of course, some other people have opinions somewhere in between, at points 2, 3, 4, 5, or 6. Where would you place yourself on this scale, or haven’t you thought much about this?

This question was reverse scored, such that 1 reflected the typical political conservatives’ position (strong preference for private health insurance), while 7 represented the typical liberal viewpoint (strong preference for government health insurance).

**Federal Spending on Welfare Programs** (two versions)

[ANES 1982]: “How would you rate the amount of federal spending on welfare? Too little, about right, or too much?” This question was coded as 0 = *too much* or *about the right amount* and 1 = *too little*. This was done to align direction of answers with other ANES waves.

[ANES 1992, 1994, 1996, 2000, 2002, 2004, 2008, 2012, 2016, 2020]: Should federal spending on welfare programs be increased, decreased, or kept the same? 1 = Welfare spending should be greatly decreased to 7 = Welfare spending should be greatly increased.

**Other ANES climate policy questions**

In addition to modeling climate policy questions for which time series are available, we also modeled all other ANES climate policy questions from 1982 – 2020. Here, we define “climate policy questions” as those asking respondents about whether action should be taken (usually by the government) to address an environmental issue, such as global warming or pollution. We exclude questions that ask about personal perceptions of climate change; we are only interested in people’s policy positions in this analysis.

[ANES 1990 & 1992] Clean Air & Water Tax: “Would you support or oppose an increase in taxes that would be used to clean up the nation's air and water?” Response options were recoded as 1 = *support* or 0 = *oppose* or *neutral*

[ANES 1990 & 1992] Enforcing Strict Pollution Standards: “Should the government force all companies to comply with strict pollution standards even if it might put some of them out of business?” Response options were recoded as 1 = *support enforcement* or 0 = *oppose* or *neutral*

[ANES 1992] Pollution Cleanup as Foreign Policy Goal: “And reducing environmental pollution around the world. Should this be a very important foreign policy goal, a somewhat important foreign policy goal, or not an important foreign policy goal at all?” Response options were recoded as 1 = *very important* or 0 = *somewhat important* or *not at all important*.

Note: ANES survey report lists this question as included in ANES 1994, but in the ANES 1994 datafile, the variable containing responses to this question is blank for all responses.

[ANES 1996] ANES asked about seven specific climate policies in this survey. All items were formatted as “Do you think the government should put less, the same amount, or more effort into: [climate policy proposal]?” The seven items were:

- Improving and protecting the environment
- Reducing air pollution
- Managing natural resources that are important to our economy, such as timber and fisheries
- Cleaning up lakes and parks for recreation such as hiking and boating
- Cleaning up hazardous or toxic waste
- Reducing solid waste and garbage
- Addressing global warming

All items were coded as binary variables, with 1 = *more government effort* and 0 = *the same amount* or *less government effort*

[ANES 2008] Fuel Standards: “Do you favor, oppose, or neither favor nor oppose the federal government requiring automakers to build cars that use less gasoline?” If *favor* or *oppose*, “Do you [favor / oppose] that a great deal, moderately, or a little?” Response options were recoded to a 7-point scale, with 1 = *oppose fuel standards a great deal* to 7 = *favor fuel standards a great deal*.

[ANES 2008] Power Plant Emission Standards: “Power plants put gases into the air that could cause global warming. Do you favor, oppose, or neither favor nor oppose the federal government lowering the amount of these gases that power plants are allowed to put into the air?” If *favor* or *oppose*, “Do you [favor / oppose] that a great deal, moderately, or a little?” Response options were recoded to a 7-point scale, with 1 = *oppose emissions regulations a great deal* to 7 = *favor emissions regulations a great deal*.

[ANES 2008] Gasoline Tax: “Do you favor, oppose, or neither favor nor oppose increasing taxes on gasoline so people either drive less or buy cars that use less gas?” If *favor* or *oppose*, “Do you [favor / oppose] that a great deal, moderately, or a little?” Response options were recoded to a 7-point scale, with 1 = *oppose increasing gasoline taxes a great deal* to 7 = *favor increasing gasoline taxes a great deal*.

[ANES 2012] Nuclear Power Plants: “Do you think the United States should have more nuclear power plants, fewer nuclear power plants, or the same number it has now?” Response options were recoded as a binary variable, with 1 = *more nuclear power plants* and 0 = *fewer* or *the same number of nuclear power plants*.

[ANES 2012] Offshore Drilling: “Do you favor, oppose, or neither favor nor oppose increased offshore drilling for oil and natural gas in U.S. waters?” Response options were recoded as a binary variable, with 1 = *favor more offshore drilling* and 0 = *neither* or *oppose more offshore drilling*.

[ANES 2016] Fracking: “‘Fracking’ is a way to drill for natural gas by pumping high pressure fluid into the ground. Do you favor, oppose, or neither favor nor oppose fracking in the U.S.?” Response options were recoded as a binary variable, with 1 = *oppose fracking* and 0 = *neither* or *favor fracking*.

[ANES 2016] Federal Action on Rising Temperatures: “Do you think the federal government should be doing more about rising temperatures, should be doing less, or is it currently doing the right amount?” If *more* or *less*, “Should it be doing a great deal [more/less], a moderate amount [more/less], or a little [more/less]?” Response options were coded with 1 = *should be doing a great deal less* to 7 = *should be doing a great deal more*.

[ANES 2020] Regulations on Greenhouse Gas Emitters: “Do you favor, oppose, or neither favor nor oppose increased government regulation on businesses that produce a great deal of greenhouse emissions linked to climate change?” If participants selected “favor” or “oppose”, a follow-up questions was asked: “Do you [favor/oppose] that a great deal, a moderate amount, or a little?” A 7-point summary variable was produced, with 7 = *favor regulations a great deal* to 1 = *oppose regulations a great deal*. This item was analyzed as a continuous variable.
