# Supplementary material for: Younger Americans are less politically polarized than older Americans about climate policies (but not about other policy domains)
Source: PLoS One. 2024 May 15;19(5):e0302434. doi: 10.1371/journal.pone.0302434 (PMC11095675; doi:10.1371/journal.pone.0302434)
Supplement: S2 Appendix — (DOCX) [file pone.0302434.s002.docx]

**S2 Appendix. Modeling details.**

For all model outputs, **bolded** entries represent models for which the Political Ideology * Age Interaction is statistically significant at *p* < 0.05, our test for differential age-based political polarization. This is our main variable of interest.

In addition to political ideology, age, and their interaction, models include four controls variables. For the control variables, household income is a continuous variable. Gender is a binary variable with reference category set to “Female”. Education is a binary variable with reference category set to “No college degree”. Education is also interacted with political ideology, as a control. Modeling rationale is discussed in Materials & Methods.

Regression coefficients marked as “-0” to indicate values that are less than 0 but round up to 0; similarly, coefficients marked as “+0” indicate values that are greater than 0 but round down to 0.
