# Supplementary material for: Younger Americans are less politically polarized than older Americans about climate policies (but not about other policy domains)
Source: PLoS One. 2024 May 15;19(5):e0302434. doi: 10.1371/journal.pone.0302434 (PMC11095675; doi:10.1371/journal.pone.0302434)
Supplement: S1 Fig — Note: Response variables are displayed with 95%-confidence interval smoothing. Years with an asterisk (e.g. 2012*) indicate significant political ideology × age interaction at p < 0.05, whereas non-asterisked survey years do not exhibit significant age-based political polarization. In all surveys, participants expressed their support for climate policies, as well as their political ideology (1 = extremely liberal to 7 = extremely conservative) and age. Models treated political ideology as a continuous variable, but for visualization purposes, people who responded 1 or 2 are coded as “liberal”, 3 or 4 or 5 are coded as “moderate”, and people who responded 6 or 7 coded as “conservative”. A listing of questions that were indexed to create each year’s climate policy support index is available in the following tables for each modeling year. (DOCX) [file pone.0302434.s003.docx]

**S1 Fig: Younger Americans have been Less Politically Polarized than Older Americans on Climate Policy Support since 2012, but not Before (Except 1990)**


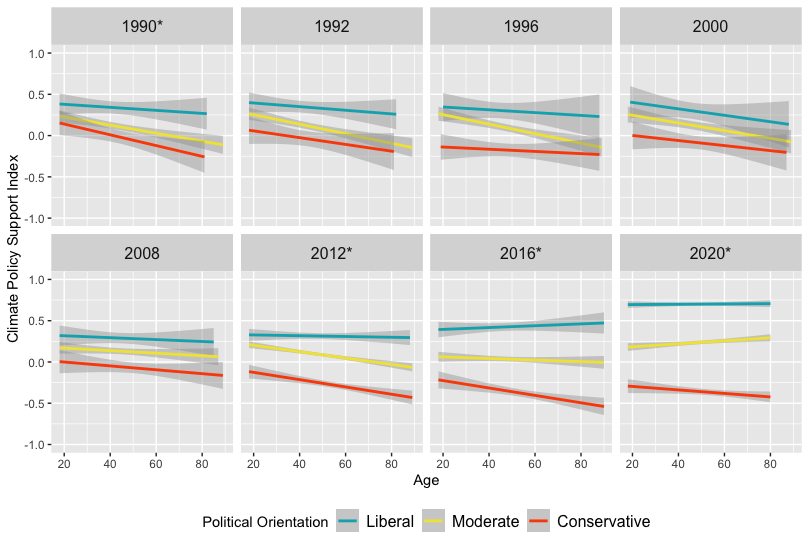


S1 Fig Note: Response variables are displayed with 95%-confidence interval smoothing. Years with an asterisk (e.g. 2012*) indicate significant political ideology × age interaction at *p* < 0.05, whereas non-asterisked survey years do not exhibit significant age-based political polarization. In all surveys, participants expressed their support for climate policies, as well as their political ideology (1 = *extremely liberal* to 7 = *extremely conservative*) and age. Models treated political ideology as a continuous variable, but for visualization purposes, people who responded 1 or 2 are coded as “liberal”, 3 or 4 or 5 are coded as “moderate”, and people who responded 6 or 7 coded as “conservative”. A listing of questions that were indexed to create each year’s climate policy support index is available in the following tables for each modeling year.
