# Supplementary material for: Younger Americans are less politically polarized than older Americans about climate policies (but not about other policy domains)
Source: PLoS One. 2024 May 15;19(5):e0302434. doi: 10.1371/journal.pone.0302434 (PMC11095675; doi:10.1371/journal.pone.0302434)
Supplement: S2 Fig — Note: Response variables are displayed with 95%-confidence interval smoothing. Years with an asterisk (e.g. 1994*) indicate significant age-based political polarization at the 0.05-significance level, whereas non-asterisked survey years do not exhibit significant age-based political polarization. In all surveys, participants expressed their support for three non-climate policies, as well as their political ideology (1 = extremely liberal to 7 = extremely conservative) and age. Models treat political ideology as a continuous variable, but for visualization purposes, people who responded 1 or 2 are coded as “liberal”, people who responded 6 or 7 coded as “conservative”, and all others as “moderate”. (DOCX) [file pone.0302434.s004.docx]

**S2 Fig: Americans Are Not Differentially Politically Polarized based on Age in Support for Defense Spending, Public Health Insurance, and Welfare Spending (Moderates Included).**


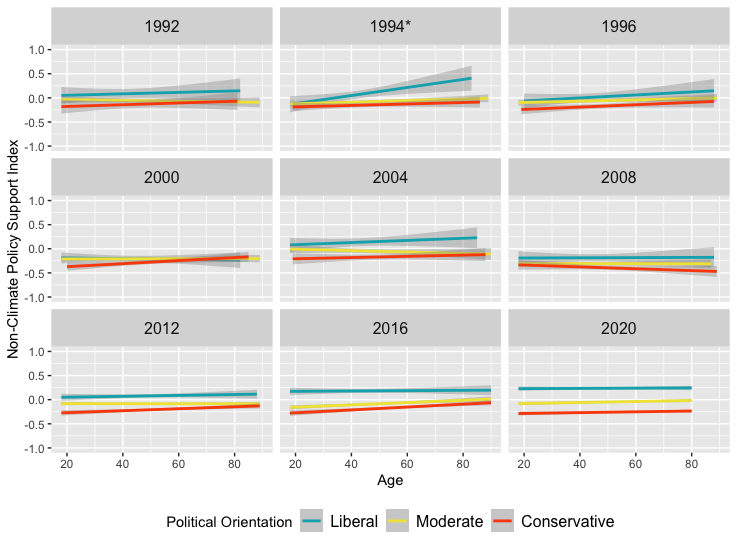


S2 Fig Note: Response variables are displayed with 95%-confidence interval smoothing. Years with an asterisk (e.g. 1994*) indicate significant age-based political polarization at the 0.05-significance level, whereas non-asterisked survey years do not exhibit significant age-based political polarization. In all surveys, participants expressed their support for three non-climate policies, as well as their political ideology (1 = *extremely liberal* to 7 = *extremely conservative*) and age. Models treat political ideology as a continuous variable, but for visualization purposes, people who responded 1 or 2 are coded as “liberal”, people who responded 6 or 7 coded as “conservative”, and all others as “moderate”.
