# Supplementary material for: Younger Americans are less politically polarized than older Americans about climate policies (but not about other policy domains)
Source: PLoS One. 2024 May 15;19(5):e0302434. doi: 10.1371/journal.pone.0302434 (PMC11095675; doi:10.1371/journal.pone.0302434)
Supplement: S1 Table — In all surveys, participants expressed their support for a range of climate policies, as well as their political ideology (1 = extremely liberal to 7 = extremely conservative) and age. Models treated political ideology as a continuous variable, but for tabulation purposes, people who responded 1 or 2 are coded as “liberal”, and people who responded 6 or 7 coded as “conservative”. For age, respondents are grouped into either “younger” (under 40 years old), “middle-aged” (40–60 years old), and “older” (over 60 years old). “% ANES Sample Retained” is the percentage of the respondents in the original ANES datafiles that we were able to model, with the remainder being excluded due to missing data. (DOCX) [file pone.0302434.s005.docx]

Supporting Information for Younger Americans are less politically polarized than older Americans about climate policies (but not about other policy domains)

**S1 Table. American National Election Studies Annual Sample Sizes, Crosstab by Political Ideology and Age Group**

| ANES Wave | Modeled Sample Size | % ANES Sample Retained | Younger Liberals | Middle-aged Liberals | Older Liberals | Younger Conservatives | Middle-aged Conservatives | Older Conservatives |
| --- | --- | --- | --- | --- | --- | --- | --- | --- |
| 1982 | 822 | 58% | 46 | 29 | 17 | 85 | 57 | 37 |
| 1984 | 1461 | 65% | 206 | 70 | 53 | 243 | 186 | 100 |
| 1986 | 841 | 39% | 101 | 31 | 17 | 149 | 99 | 58 |
| 1988 | 1292 | 63% | 80 | 35 | 25 | 122 | 96 | 81 |
| 1990 | 1206 | 61% | 92 | 38 | 31 | 84 | 73 | 47 |
| 1992 | 878 | 35% | 65 | 28 | 21 | 61 | 53 | 34 |
| 1994 | 1273 | 71% | 62 | 55 | 14 | 148 | 117 | 84 |
| 1996 | 1136 | 66% | 63 | 54 | 28 | 112 | 99 | 71 |
| 1998 | 956 | 75% | 49 | 48 | 13 | 60 | 75 | 49 |
| 2000 | 1434 | 79% | 94 | 86 | 38 | 110 | 147 | 86 |
| 2002 | 1164 | 77% | 60 | 90 | 43 | 94 | 140 | 108 |
| 2004 | 825 | 68% | 58 | 48 | 23 | 58 | 88 | 61 |
| 2008 | 1494 | 64% | 117 | 109 | 48 | 87 | 156 | 104 |
| 2012 | 5058 | 86% | 270 | 319 | 208 | 223 | 460 | 469 |
| 2016 | 3096 | 73% | 266 | 178 | 176 | 173 | 271 | 360 |
| 2020 | 6531 | 79% | 578 | 428 | 491 | 370 | 551 | 833 |

S1 Table Legend: In all surveys, participants expressed their support for a range of climate policies, as well as their political ideology (1 = extremely liberal to 7 = extremely conservative) and age. Models treated political ideology as a continuous variable, but for tabulation purposes, people who responded 1 or 2 are coded as “liberal”, and people who responded 6 or 7 coded as “conservative”. For age, respondents are grouped into either “younger” (under 40 years old), “middle-aged” (40 – 60 years old), and “older” (over 60 years old). “% ANES Sample Retained” is the percentage of the respondents in the original ANES datafiles that we were able to model, with the remainder being excluded due to missing data.
