# Supplementary material for: Younger Americans are less politically polarized than older Americans about climate policies (but not about other policy domains)
Source: PLoS One. 2024 May 15;19(5):e0302434. doi: 10.1371/journal.pone.0302434 (PMC11095675; doi:10.1371/journal.pone.0302434)
Supplement: S2 Table — (DOCX) [file pone.0302434.s006.docx]

**S2 Table. ANES Environment and Climate Policy Questions and Response Scales (Non-time-series Items)**

| **Climate Policy Question** | **Response Scale: Lower Endpoint vs Upper Endpoint** | **ANES Wave** |
| --- | --- | --- |
| Clean Air & Water Tax | 0 = *Oppose* or *neutral* to 1 = *Support* | 1990, 1992 |
| Enforcing Strict Pollution Standards | 0 = *Oppose* or *neutral* to 1 = *Support* | 1990, 1992 |
| Pollution Cleanup as Foreign Policy Goal | 0 = *Somewhat important* or *not very important* to 1 = *Very important* | 1992 |
| Improve and Protect the Environment | 0 = *Less* or *the same amount of government effort* to 1 = *More government effort* | 1996 |
| Reduce Air Pollution | 0 = *Less* or *the same amount of government effort* to 1 = *More government effort* | 1996 |
| Manage Natural Resources | 0 = *Less* or *the same amount of government effort* to 1 = *More government effort* | 1996 |
| Clean up Lakes and Parks | 0 = *Less* or *the same amount of government effort* to 1 = *More government effort* | 1996 |
| Clean up Toxic Waste | 0 = *Less* or *the same amount of government effort* to 1 = *More government effort* | 1996 |
| Reduce Garbage | 0 = *Less* or *the same amount of government effort* to 1 = *More government effort* | 1996 |
| Address Global Warming | 0 = *Less* or *the same amount of government effort* to 1 = *More government effort* | 1996 |
| Fuel Standards | 1 = *Oppose emissions regulations a great deal* to 7 = *Favor emissions regulations a great deal* | 2008 |
| Power Plant Emission Standards | 1 = *Oppose emissions regulations a great deal* to 7 = *Favor emissions regulations a great deal* | 2008 |
| Gasoline Tax | 1 = *Oppose emissions regulations a great deal* to 7 = *Favor emissions regulations a great deal* | 2008 |
| Nuclear Power Plants | 0 = *More* or *the same number of nuclear power plants* to 1 = *Fewer nuclear power plants* | 2012 |
| Offshore Drilling | 0 = *Favor* or *neither favor nor oppose* to 1 = *Oppose* | 2012 |
| Fracking | 0 = *Favor or neither favor nor oppose* to 1 = *Oppose* | 2016 |
| Federal Action on Rising Temperatures | 1 = *Should be doing a great deal less* to 7 = *Should be doing a great deal more* | 2016 |
| Regulations on Greenhouse Gas Emitters | 1 = *Oppose regulations a great deal* to 7 = *Favor regulations a great deal* | 2020 |
