# Supplementary material for: Younger Americans are less politically polarized than older Americans about climate policies (but not about other policy domains)
Source: PLoS One. 2024 May 15;19(5):e0302434. doi: 10.1371/journal.pone.0302434 (PMC11095675; doi:10.1371/journal.pone.0302434)
Supplement: S3 Table — (DOCX) [file pone.0302434.s007.docx]

**S3 Table. Regression model for federal spending on the environment survey question (ANES 2020; logistic regression).**

| Variable | Standardized Coefficient (Cohen’s *d*) | Standardized 95% Confidence Interval | *p*-value | Unstandardized Coefficient |
| --- | --- | --- | --- | --- |
| Political Ideology | -1.318 | [-1.437, -1.202] | < 0.001 | -0.548 |
| Age | -0 | [-0.063, 0.063] | 0.002 | 0.019 |
| Political Ideology * Age Interaction | **-0.133** | **[-0.207, -0.06]** | **< 0.001** | -0.005 |
| Gender (Male) | -0.03 | [-0.15, 0.091] | 0.626 | -0.03 |
| Household Income | -0.024 | [-0.087, 0.039] | 0.46 | -0 |
| Education (College Degree) | 0.211 | [0.079, 0.343] | 0.001 | 0.719 |
| Political Ideology * Education (College Degree) Interaction | -0.209 | [-0.36, -0.057] | 0.007 | -0.125 |
| Intercept | 0.501 | [0.384, 0.62] | < 0.001 | 2.762 |
| Model statistics: *n* = 6,517; McFadden's pseudo-R^2^ = 0.25.  Survey question: “What about protecting the environment – should federal spending on protecting the environment be increased, decreased, or kept the same?”  Response coding: *Increase* = 1, all other responses = 0. | | | | |
