# Supplementary material for: Younger Americans are less politically polarized than older Americans about climate policies (but not about other policy domains)
Source: PLoS One. 2024 May 15;19(5):e0302434. doi: 10.1371/journal.pone.0302434 (PMC11095675; doi:10.1371/journal.pone.0302434)
Supplement: S4 Table — (DOCX) [file pone.0302434.s008.docx]

**S4 Table. Regression model for environment regulations vs business interests survey question (ANES 2020; linear regression).**

| Variable | Standardized Coefficient (Cohen’s *d*) | Standardized 95% Confidence Interval | *p*-value | Unstandardized Coefficient |
| --- | --- | --- | --- | --- |
| Political Ideology | -0.625 | [-0.658, -0.591] | < 0.001 | -0.549 |
| Age | -0.064 | [-0.084, -0.045] | 0.017 | 0.007 |
| Political Ideology * Age Interaction | **-0.052** | **[-0.071, -0.033]** | **< 0.001** | -0.004 |
| Gender (Male) | -0.095 | [-0.134, -0.056] | < 0.001 | -0.189 |
| Household Income | -0.005 | [-0.025, 0.015] | 0.623 | -0 |
| Education (College Degree) Interaction | 0.015 | [-0.027, 0.057] | 0.399 | 0.094 |
| Political Ideology * Education (College Degree) Interaction | -0.013 | [-0.054, 0.028] | 0.52 | -0.016 |
| Intercept | 0.045 | [0.006, 0.084] | < 0.001 | 7.615 |
| Model statistics: *n* = 5,919; multiple R^2^ = 0.43.  Survey question: “Where would you place yourself on this scale, or haven’t you thought much about this? 1 = *Tougher regulations on business needed to protect environment* to 7 = *Regulations to protect environment already too much a burden on business.*”  Response coding: Reverse-coded, ranging from 1 = *Regulations to protect environment already too much a burden on business* to 7 = *Tougher regulations on business needed to protect environment.* | | | | |
