# Supplementary material for: Younger Americans are less politically polarized than older Americans about climate policies (but not about other policy domains)
Source: PLoS One. 2024 May 15;19(5):e0302434. doi: 10.1371/journal.pone.0302434 (PMC11095675; doi:10.1371/journal.pone.0302434)
Supplement: S5 Table — (DOCX) [file pone.0302434.s009.docx]

**S5 Table. Regression model for regulations on greenhouse gas emitters survey question (ANES 2020; linear regression).**

| Variable | Standardized Coefficient (Cohen’s *d*) | Standardized 95% Confidence Interval | *p*-value | Unstandardized Coefficient |
| --- | --- | --- | --- | --- |
| Political Ideology | -0.502 | [-0.538, -0.465] | < 0.001 | -0.386 |
| Age | 0.048 | [0.027, 0.069] | < 0.001 | 0.019 |
| Political Ideology * Age Interaction | **-0.053** | **[-0.074, -0.032]** | **< 0.001** | -0.003 |
| Gender (Male) | -0.082 | [-0.125, -0.04] | < 0.001 | -0.156 |
| Household Income | 0.028 | [0.006, 0.05] | 0.013 | +0 |
| Education (College Degree) Interaction | 0.075 | [0.029, 0.12] | < 0.001 | 0.511 |
| Political Ideology * Education (College Degree) Interaction | -0.081 | [-0.125, -0.036] | < 0.001 | -0.091 |
| Intercept | -0.005 | [-0.047, 0.037] | < 0.001 | 6.501 |
| Model statistics: *n* = 5,900; multiple R^2^ = 0.32.  Survey question: “Do you favor, oppose, or neither favor nor oppose increased government regulation on businesses that produce a great deal of greenhouse emissions linked to climate change?”  Response coding: Ranges from 1 = *Oppose regulations a great deal* to 7 = *Favor regulations a great deal.* | | | | |
