# Supplementary material for: Younger Americans are less politically polarized than older Americans about climate policies (but not about other policy domains)
Source: PLoS One. 2024 May 15;19(5):e0302434. doi: 10.1371/journal.pone.0302434 (PMC11095675; doi:10.1371/journal.pone.0302434)
Supplement: S6 Table — (DOCX) [file pone.0302434.s010.docx]

**S6 Table. Regression model for climate policy support index in ANES 1990 (three-item index; linear regression).**

| Variable | Standardized Coefficient (Cohen’s *d*) | Standardized 95% Confidence Interval | *p*-value | Unstandardized Coefficient |
| --- | --- | --- | --- | --- |
| Political Ideology | -0.17 | [-0.24, -0.101] | 0.873 | -0.007 |
| Age | -0.143 | [-0.197, -0.089] | 0.624 | 0.002 |
| Political Ideology * Age Interaction | **-0.06** | **[-0.117, -0.004]** | **0.036** | -0.002 |
| Gender (Male) | 0.069 | [-0.04, 0.179] | 0.214 | 0.049 |
| Household Income | -0.044 | [-0.101, 0.014] | 0.136 | -0 |
| Education (College Degree) Interaction | 0.129 | [0.006, 0.251] | 0.002 | 0.395 |
| Political Ideology * Education (College Degree) Interaction | -0.139 | [-0.251, -0.026] | 0.016 | -0.073 |
| Intercept | -0.081 | [-0.167, 0.005] | 0.154 | 0.271 |
| Model statistics: *n* = 1,206; multiple R^2^ = 0.09.  Constituent survey questions: Federal spending on the environment, clean air & water tax, and enforce pollution regulations.  The index was formed by averaging normalized responses to the constituent survey questions. Higher index scores reflect greater preference for climate policies. | | | | |
