# Supplementary material for: Younger Americans are less politically polarized than older Americans about climate policies (but not about other policy domains)
Source: PLoS One. 2024 May 15;19(5):e0302434. doi: 10.1371/journal.pone.0302434 (PMC11095675; doi:10.1371/journal.pone.0302434)
Supplement: S7 Table — (DOCX) [file pone.0302434.s011.docx]

**S7 Table. Regression model for climate policy support index in ANES 1992 (four-item index; linear regression).**

| Variable | Standardized Coefficient (Cohen’s *d*) | Standardized 95% Confidence Interval | *p*-value | Unstandardized Coefficient |
| --- | --- | --- | --- | --- |
| Political Ideology | -0.21 | [-0.294, -0.127] | 0.503 | -0.031 |
| Age | -0.156 | [-0.22, -0.093] | 0.87 | 0.001 |
| Political Ideology * Age Interaction | -0.056 | [-0.123, 0.011] | 0.103 | -0.002 |
| Gender (Male) | -0.008 | [-0.136, 0.119] | 0.896 | -0.005 |
| Household Income | -0.04 | [-0.109, 0.028] | 0.248 | -0 |
| Education (College Degree) Interaction | 0.073 | [-0.07, 0.216] | 0.034 | 0.292 |
| Political Ideology * Education (College Degree) Interaction | -0.122 | [-0.251, 0.007] | 0.064 | -0.059 |
| Intercept | -0.025 | [-0.129, 0.078] | 0.05 | 0.408 |
| Model statistics: *n* = 878; multiple R^2^ = 0.11.  Constituent survey questions: Federal spending on the environment, clean air & water tax, enforce pollution regulations, and pollution cleanup as foreign policy goal.  The index was formed by averaging normalized responses to the constituent survey questions. Higher index scores reflect greater preference for climate policies. | | | | |
