# Supplementary material for: Younger Americans are less politically polarized than older Americans about climate policies (but not about other policy domains)
Source: PLoS One. 2024 May 15;19(5):e0302434. doi: 10.1371/journal.pone.0302434 (PMC11095675; doi:10.1371/journal.pone.0302434)
Supplement: S14 Table — (DOCX) [file pone.0302434.s018.docx]

**S14 Table: Annual regression models for federal spending on the environment ANES time-series (logistic regressions).**

| ANES Year | Standardized Political Ideology * Age Interaction Coefficient (Cohen’s *d*) | Standardized 95% Confidence Interval | *p*-value | Sample Size | McFadden's pseudo-R^2^ |
| --- | --- | --- | --- | --- | --- |
| 1982 | -0.004 | [-0.163, 0.154] | 0.96 | 779 | 0.07 |
| 1984 | -0.053 | [-0.168, 0.06] | 0.36 | 1416 | 0.04 |
| 1986 | -0.018 | [-0.164, 0.126] | 0.8 | 829 | 0.03 |
| 1988 | -0.036 | [-0.154, 0.082] | 0.55 | 1283 | 0.04 |
| 1990 | -0.023 | [-0.153, 0.106] | 0.73 | 1192 | 0.07 |
| 1992 | -0.037 | [-0.195, 0.12] | 0.64 | 871 | 0.08 |
| 1994 | 0.004 | [-0.133, 0.14] | 0.95 | 1262 | 0.07 |
| 1996 | 0.083 | [-0.048, 0.213] | 0.22 | 1133 | 0.05 |
| 1998 | NA |  |  |  |  |
| 2000 | **-0.143** | **[-0.262, -0.025]** | **0.02** | 1419 | 0.06 |
| 2002 | 0.033 | [-0.101, 0.166] | 0.63 | 1045 | 0.08 |
| 2004 | NA |  |  |  |  |
| 2008 | -0.055 | [-0.175, 0.063] | 0.36 | 1480 | 0.06 |
| 2012 | **-0.135** | **[-0.201, -0.069]** | **< 0.001** | 5044 | 0.1 |
| 2016 | **-0.116** | **[-0.201, -0.03]** | **0.01** | 3089 | 0.17 |
| 2020 | **-0.133** | **[-0.207, -0.06]** | **< 0.001** | 6517 | 0.25 |
| Typical survey question wording: “What about protecting the environment – should federal spending on protecting the environment be increased, decreased, or kept the same?” This survey question was not asked in 1998 or 2004. Year-to-year question wording is available in the question wording and coding decisions section in Supporting Information.  Response coding: *Increase* = 1, all other responses = 0.  Models controlled for political ideology, age, education, the interaction between education and political ideology, gender and household income. | | | | | |
