# Supplementary material for: Younger Americans are less politically polarized than older Americans about climate policies (but not about other policy domains)
Source: PLoS One. 2024 May 15;19(5):e0302434. doi: 10.1371/journal.pone.0302434 (PMC11095675; doi:10.1371/journal.pone.0302434)
Supplement: S15 Table — (DOCX) [file pone.0302434.s019.docx]

**S15 Table: Annual regression models for environmental regulations versus business interests ANES time-series (linear regressions).**

| ANES Year | Standardized Political Ideology * Age Interaction Coefficient (Cohen’s *d*) | Standardized 95% Confidence Interval | *p*-value | Sample Size | Multiple R^2^ |
| --- | --- | --- | --- | --- | --- |
| 1996 | 0.027 | [-0.032, 0.085] | 0.37 | 947 | 0.2 |
| 1998 | -0.03 | [-0.096, 0.036] | 0.37 | 856 | 0.13 |
| 2000 | 0.049 | [-0.011, 0.109] | 0.11 | 1046 | 0.1 |
| 2002 | NA |  |  |  |  |
| 2004 | -0.051 | [-0.118, 0.016] | 0.14 | 750 | 0.13 |
| 2008 | -0.045 | [-0.117, 0.028] | 0.23 | 659 | 0.13 |
| 2012 | **-0.065** | **[-0.091, -0.039]** | **< 0.001** | 4419 | 0.24 |
| 2016 | **-0.056** | **[-0.087, -0.024]** | **< 0.001** | 2754 | 0.28 |
| 2020 | **-0.052** | **[-0.071, -0.033]** | **< 0.001** | 5919 | 0.43 |
| Typical survey question wording: “Some people think we need much tougher government regulations on business in order to protect the environment. Suppose these people are at one end of a scale, at point 1. Others think that current regulations to protect the environment are already too much of a burden on business. Suppose these people are at the other end, at point 7. And, of course, some other people have opinions somewhere in between, at points 2,3,4,5, or 6. Where would you place yourself on this scale, or haven’t you thought much about this?” This question was not asked before 1996, and it was not asked in 2002. Year-to-year question wording is available in the question wording and coding decisions section in Supporting Information.  Response coding: *Increase* = 1, all other responses = 0.  Models controlled for political ideology, age, education, the interaction between education and political ideology, gender and household income. | | | | | |
