# Supplementary material for: Younger Americans are less politically polarized than older Americans about climate policies (but not about other policy domains)
Source: PLoS One. 2024 May 15;19(5):e0302434. doi: 10.1371/journal.pone.0302434 (PMC11095675; doi:10.1371/journal.pone.0302434)
Supplement: S16 Table — (DOCX) [file pone.0302434.s020.docx]

**S16 Table. Regression model for clean air & water tax survey question (ANES 1990; logistic regression).**

| Variable | Standardized Coefficient (Cohen’s *d*) | Standardized 95% Confidence Interval | *p*-value | Unstandardized Coefficient |
| --- | --- | --- | --- | --- |
| Political Ideology | -0.343 | [-0.51, -0.18] | 0.531 | -0.096 |
| Age | -0.212 | [-0.338, -0.087] | 0.84 | 0.003 |
| Political Ideology * Age Interaction | -0.083 | [-0.22, 0.053] | 0.235 | -0.004 |
| Gender (Male) | 0.085 | [-0.168, 0.339] | 0.511 | 0.085 |
| Household Income | -0.07 | [-0.202, 0.063] | 0.3 | -0 |
| Education (College Degree) Interaction | 0.045 | [-0.241, 0.335] | 0.058 | 0.922 |
| Political Ideology * Education (College Degree) Interaction | -0.279 | [-0.562, -0.002] | 0.05 | -0.21 |
| Intercept | 0.715 | [0.519, 0.915] | 0.01 | 1.768 |
| Model statistics: *n* = 1,187; McFadden’s pseudo-R^2^ = 0.05.  Survey question: “Would you support or oppose an increase in taxes that would be used to clean up the nation's air and water?”  Response coding: *Support* = 1, all other responses = 0. | | | | |
