# Supplementary material for: Younger Americans are less politically polarized than older Americans about climate policies (but not about other policy domains)
Source: PLoS One. 2024 May 15;19(5):e0302434. doi: 10.1371/journal.pone.0302434 (PMC11095675; doi:10.1371/journal.pone.0302434)
Supplement: S17 Table — (DOCX) [file pone.0302434.s021.docx]

**S17 Table. Regression model for enforcing strict pollution regulations survey question (ANES 1990; logistic regression).**

| Variable | Standardized Coefficient (Cohen’s *d*) | Standardized 95% Confidence Interval | *p*-value | Unstandardized Coefficient |
| --- | --- | --- | --- | --- |
| Political Ideology | -0.205 | [-0.406, -0.008] | 0.298 | 0.19 |
| Age | 0.039 | [-0.121, 0.204] | 0.05 | 0.035 |
| Political Ideology * Age Interaction | **-0.175** | **[-0.346, -0.006]** | **0.044** | -0.008 |
| Gender (Male) | 0.1 | [-0.213, 0.414] | 0.53 | 0.1 |
| Household Income | -0.154 | [-0.311, 0.005] | 0.055 | -0 |
| Education (College Degree) Interaction | 0.198 | [-0.154, 0.561] | 0.083 | 1.036 |
| Political Ideology * Education (College Degree) Interaction | -0.267 | [-0.61, 0.069] | 0.122 | -0.201 |
| Intercept | 1.537 | [1.301, 1.783] | 0.296 | 0.864 |
| Model statistics: *n* = 1,183; McFadden’s pseudo-R^2^ = 0.02.  Survey question: “Should the government force all companies to comply with strict pollution standards even if it might put some of them out of business?”  Response coding: *Support* *enforcements* = 1, all other responses = 0. | | | | |
