# Supplementary material for: Younger Americans are less politically polarized than older Americans about climate policies (but not about other policy domains)
Source: PLoS One. 2024 May 15;19(5):e0302434. doi: 10.1371/journal.pone.0302434 (PMC11095675; doi:10.1371/journal.pone.0302434)
Supplement: S18 Table — (DOCX) [file pone.0302434.s022.docx]

**S18 Table. Regression model for clean air & water tax survey question (ANES 1992; logistic regression).**

| Variable | Standardized Coefficient (Cohen’s *d*) | Standardized 95% Confidence Interval | *p*-value | Unstandardized Coefficient |
| --- | --- | --- | --- | --- |
| Political Ideology | -0.426 | [-0.63, -0.228] | 0.337 | -0.173 |
| Age | -0.263 | [-0.409, -0.117] | 0.918 | -0.002 |
| Political Ideology * Age Interaction | -0.075 | [-0.239, 0.086] | 0.365 | -0.003 |
| Gender (Male) | 0.022 | [-0.277, 0.321] | 0.884 | 0.022 |
| Household Income | 0.052 | [-0.109, 0.216] | 0.529 | +0 |
| Education (College Degree) Interaction | 0.118 | [-0.22, 0.459] | 0.66 | 0.242 |
| Political Ideology * Education (College Degree) Interaction | -0.04 | [-0.36, 0.276] | 0.806 | -0.03 |
| Intercept | 0.718 | [0.478, 0.965] | 0.013 | 2.05 |
| Model statistics: *n* = 864; McFadden’s pseudo-R^2^ = 0.05.  Survey question: “Would you support or oppose an increase in taxes that would be used to clean up the nation's air and water?”  Response coding: *Support* = 1, all other responses = 0. | | | | |
