# Supplementary material for: Younger Americans are less politically polarized than older Americans about climate policies (but not about other policy domains)
Source: PLoS One. 2024 May 15;19(5):e0302434. doi: 10.1371/journal.pone.0302434 (PMC11095675; doi:10.1371/journal.pone.0302434)
Supplement: S19 Table — (DOCX) [file pone.0302434.s023.docx]

**S19 Table. Regression model for enforcing strict pollution regulations survey question (ANES 1992; logistic regression).**

| Variable | Standardized Coefficient (Cohen’s *d*) | Standardized 95% Confidence Interval | *p*-value | Unstandardized Coefficient |
| --- | --- | --- | --- | --- |
| Political Ideology | -0.096 | [-0.333, 0.14] | 0.308 | 0.216 |
| Age | 0.038 | [-0.144, 0.225] | 0.148 | 0.03 |
| Political Ideology * Age Interaction | -0.147 | [-0.347, 0.05] | 0.147 | -0.007 |
| Gender (Male) | 0.063 | [-0.3, 0.426] | 0.734 | 0.063 |
| Household Income | -0.13 | [-0.316, 0.06] | 0.173 | -0 |
| Education (College Degree) Interaction | 0.111 | [-0.293, 0.525] | 0.056 | 1.263 |
| Political Ideology * Education (College Degree) Interaction | -0.368 | [-0.749, 0.005] | 0.055 | -0.276 |
| Intercept | 1.519 | [1.237, 1.815] | 0.461 | 0.702 |
| Model statistics: *n* = 865; McFadden’s pseudo-R^2^ = 0.02.  Survey question: “Should the government force all companies to comply with strict pollution standards even if it might put some of them out of business?”  Response coding: *Support* *enforcements* = 1, all other responses = 0. | | | | |
