# Supplementary material for: Younger Americans are less politically polarized than older Americans about climate policies (but not about other policy domains)
Source: PLoS One. 2024 May 15;19(5):e0302434. doi: 10.1371/journal.pone.0302434 (PMC11095675; doi:10.1371/journal.pone.0302434)
Supplement: S20 Table — (DOCX) [file pone.0302434.s024.docx]

**S20 Table. Regression model for pollution cleanup as a foreign policy goal survey question (ANES 1992; logistic regression).**

| Variable | Standardized Coefficient (Cohen’s *d*) | Standardized 95% Confidence Interval | *p*-value | Unstandardized Coefficient |
| --- | --- | --- | --- | --- |
| Political Ideology | -0.335 | [-0.562, -0.115] | 0.248 | -0.233 |
| Age | -0.194 | [-0.358, -0.029] | 0.595 | -0.01 |
| Political Ideology * Age Interaction | -0.01 | [-0.187, 0.166] | 0.913 | -0 |
| Gender (Male) | -0.123 | [-0.452, 0.206] | 0.465 | -0.123 |
| Household Income | -0.171 | [-0.341, 0.002] | 0.051 | -0 |
| Education (College Degree) Interaction | -0.211 | [-0.579, 0.16] | 0.419 | 0.496 |
| Political Ideology * Education (College Degree) Interaction | -0.225 | [-0.575, 0.12] | 0.205 | -0.169 |
| Intercept | 1.243 | [0.975, 1.522] | 0.002 | 2.964 |
| Model statistics: *n* = 814; McFadden’s pseudo-R^2^ = 0.05.  Survey question: “And reducing environmental pollution around the world. Should this be a very important foreign policy goal, a somewhat important foreign policy goal, or not an important foreign policy goal at all?  Response coding: 1 = *very important,* 0 = *somewhat important* or *not at all important.* | | | | |
