# Supplementary material for: Younger Americans are less politically polarized than older Americans about climate policies (but not about other policy domains)
Source: PLoS One. 2024 May 15;19(5):e0302434. doi: 10.1371/journal.pone.0302434 (PMC11095675; doi:10.1371/journal.pone.0302434)
Supplement: S21 Table — (DOCX) [file pone.0302434.s025.docx]

**S21 Table. Regression model for improving and protecting the environment survey question (ANES 1996; logistic regression).**

| Variable | Standardized Coefficient (Cohen’s *d*) | Standardized 95% Confidence Interval | *p*-value | Unstandardized Coefficient |
| --- | --- | --- | --- | --- |
| Political Ideology | -0.567 | [-0.753, -0.387] | < 0.001 | -0.618 |
| Age | -0.251 | [-0.377, -0.125] | 0.008 | -0.035 |
| Political Ideology * Age Interaction | 0.11 | [-0.025, 0.245] | 0.108 | 0.005 |
| Gender (Male) | 0.004 | [-0.248, 0.257] | 0.975 | 0.004 |
| Household Income | -0.052 | [-0.188, 0.084] | 0.452 | -0 |
| Education (College Degree) Interaction | -0.259 | [-0.532, 0.014] | 0.665 | 0.196 |
| Political Ideology * Education (College Degree) Interaction | -0.149 | [-0.42, 0.121] | 0.28 | -0.106 |
| Intercept | 0.294 | [0.093, 0.496] | < 0.001 | 3.733 |
| Model statistics: *n* = 1,132; McFadden’s pseudo-R^2^ = 0.08.  Survey question: “Do you think the government should put less, the same amount, or more effort into: Improving and protecting the environment?”  Response coding: 1 = *more government effort,* 0 = *the same amount* or *less government effort.* | | | | |
