# Supplementary material for: Younger Americans are less politically polarized than older Americans about climate policies (but not about other policy domains)
Source: PLoS One. 2024 May 15;19(5):e0302434. doi: 10.1371/journal.pone.0302434 (PMC11095675; doi:10.1371/journal.pone.0302434)
Supplement: S22 Table — (DOCX) [file pone.0302434.s026.docx]

**S22 Table. Regression model for reducing air pollution survey question (ANES 1996; logistic regression).**

| Variable | Standardized Coefficient (Cohen’s *d*) | Standardized 95% Confidence Interval | *p*-value | Unstandardized Coefficient |
| --- | --- | --- | --- | --- |
| Political Ideology | -0.508 | [-0.692, -0.33] | 0.026 | -0.348 |
| Age | -0.114 | [-0.24, 0.012] | 0.669 | -0.006 |
| Political Ideology * Age Interaction | -0.006 | [-0.142, 0.129] | 0.934 | -0 |
| Gender (Male) | -0.217 | [-0.469, 0.036] | 0.092 | -0.217 |
| Household Income | -0.023 | [-0.159, 0.113] | 0.738 | -0 |
| Education (College Degree) Interaction | -0.204 | [-0.478, 0.07] | 0.288 | 0.486 |
| Political Ideology * Education (College Degree) Interaction | -0.226 | [-0.498, 0.043] | 0.101 | -0.16 |
| Intercept | 0.555 | [0.353, 0.76] | 0.001 | 2.407 |
| Model statistics: *n* = 1,133; McFadden’s pseudo-R^2^ = 0.07.  Survey question: “Do you think the government should put less, the same amount, or more effort into: reducing air pollution?”  Response coding: 1 = *more government effort,* 0 = *the same amount* or *less government effort.* | | | | |
