# Supplementary material for: Younger Americans are less politically polarized than older Americans about climate policies (but not about other policy domains)
Source: PLoS One. 2024 May 15;19(5):e0302434. doi: 10.1371/journal.pone.0302434 (PMC11095675; doi:10.1371/journal.pone.0302434)
Supplement: S23 Table — (DOCX) [file pone.0302434.s027.docx]

**S23 Table. Regression model for managing natural resources survey question (ANES 1996; logistic regression).**

| Variable | Standardized Coefficient (Cohen’s *d*) | Standardized 95% Confidence Interval | *p*-value | Unstandardized Coefficient |
| --- | --- | --- | --- | --- |
| Political Ideology | -0.521 | [-0.705, -0.342] | 0.009 | -0.402 |
| Age | -0.118 | [-0.243, 0.007] | 0.442 | -0.01 |
| Political Ideology * Age Interaction | 0.017 | [-0.116, 0.148] | 0.805 | 0.001 |
| Gender (Male) | 0.165 | [-0.086, 0.416] | 0.198 | 0.165 |
| Household Income | -0.046 | [-0.179, 0.088] | 0.499 | -0 |
| Education (College Degree) Interaction | -0.287 | [-0.556, -0.018] | 0.504 | -0.295 |
| Political Ideology * Education (College Degree) Interaction | 0.003 | [-0.258, 0.264] | 0.984 | 0.002 |
| Intercept | 0.369 | [0.169, 0.57] | < 0.001 | 2.5 |
| Model statistics: *n* = 1,124; McFadden’s pseudo-R^2^ = 0.05.  Survey question: “Do you think the government should put less, the same amount, or more effort into: Managing natural resources that are important to our economy, such as timber and fisheries?”  Response coding: 1 = *more government effort,* 0 = *the same amount* or *less government effort.* | | | | |
