# Supplementary material for: Younger Americans are less politically polarized than older Americans about climate policies (but not about other policy domains)
Source: PLoS One. 2024 May 15;19(5):e0302434. doi: 10.1371/journal.pone.0302434 (PMC11095675; doi:10.1371/journal.pone.0302434)
Supplement: S24 Table — (DOCX) [file pone.0302434.s028.docx]

**S24 Table. Regression model for cleaning up lakes and parks survey question (ANES 1996; logistic regression).**

| Variable | Standardized Coefficient (Cohen’s *d*) | Standardized 95% Confidence Interval | *p*-value | Unstandardized Coefficient |
| --- | --- | --- | --- | --- |
| Political Ideology | -0.272 | [-0.443, -0.104] | 0.523 | -0.092 |
| Age | -0.1 | [-0.221, 0.021] | 0.783 | 0.003 |
| Political Ideology * Age Interaction | -0.051 | [-0.177, 0.074] | 0.423 | -0.002 |
| Gender (Male) | 0.2 | [-0.043, 0.444] | 0.108 | 0.2 |
| Household Income | -0.104 | [-0.234, 0.026] | 0.118 | -0 |
| Education (College Degree) Interaction | -0.416 | [-0.677, -0.157] | 0.36 | -0.372 |
| Political Ideology * Education (College Degree) Interaction | -0.014 | [-0.259, 0.23] | 0.908 | -0.01 |
| Intercept | 0.195 | [0.002, 0.39] | 0.113 | 1.022 |
| Model statistics: *n* = 1,131; McFadden’s pseudo-R^2^ = 0.03.  Survey question: “Do you think the government should put less, the same amount, or more effort into: Cleaning up lakes and parks for recreation such as hiking and boating?”  Response coding: 1 = *more government effort,* 0 = *the same amount* or *less government effort.* | | | | |
