# Supplementary material for: Younger Americans are less politically polarized than older Americans about climate policies (but not about other policy domains)
Source: PLoS One. 2024 May 15;19(5):e0302434. doi: 10.1371/journal.pone.0302434 (PMC11095675; doi:10.1371/journal.pone.0302434)
Supplement: S25 Table — (DOCX) [file pone.0302434.s029.docx]

**S25 Table. Regression model for cleaning up hazardous or toxic waste survey question (ANES 1996; logistic regression).**

| Variable | Standardized Coefficient (Cohen’s *d*) | Standardized 95% Confidence Interval | *p*-value | Unstandardized Coefficient |
| --- | --- | --- | --- | --- |
| Political Ideology | -0.322 | [-0.53, -0.118] | 0.172 | -0.232 |
| Age | -0.119 | [-0.259, 0.021] | 0.607 | -0.007 |
| Political Ideology * Age Interaction | 0.002 | [-0.141, 0.145] | 0.976 | +0 |
| Gender (Male) | -0.31 | [-0.583, -0.036] | 0.026 | -0.31 |
| Household Income | -0.054 | [-0.197, 0.091] | 0.465 | -0 |
| Education (College Degree) Interaction | -0.615 | [-0.911, -0.321] | 0.405 | -0.405 |
| Political Ideology * Education (College Degree) Interaction | -0.069 | [-0.351, 0.213] | 0.632 | -0.049 |
| Intercept | 1.433 | [1.2, 1.675] | < 0.001 | 2.843 |
| Model statistics: *n* = 1,128; McFadden’s pseudo-R^2^ = 0.05.  Survey question: “Do you think the government should put less, the same amount, or more effort into: Cleaning up hazardous or toxic waste?”  Response coding: 1 = *more government effort,* 0 = *the same amount* or *less government effort.* | | | | |
