# Supplementary material for: Younger Americans are less politically polarized than older Americans about climate policies (but not about other policy domains)
Source: PLoS One. 2024 May 15;19(5):e0302434. doi: 10.1371/journal.pone.0302434 (PMC11095675; doi:10.1371/journal.pone.0302434)
Supplement: S26 Table — (DOCX) [file pone.0302434.s030.docx]

**S26 Table. Regression model for reducing solid waste and garbage survey question (ANES 1996; logistic regression).**

| Variable | Standardized Coefficient (Cohen’s *d*) | Standardized 95% Confidence Interval | *p*-value | Unstandardized Coefficient |
| --- | --- | --- | --- | --- |
| Political Ideology | -0.295 | [-0.472, -0.122] | 0.59 | -0.08 |
| Age | -0.092 | [-0.216, 0.032] | 0.611 | 0.006 |
| Political Ideology * Age Interaction | -0.066 | [-0.195, 0.062] | 0.316 | -0.003 |
| Gender (Male) | -0.14 | [-0.389, 0.109] | 0.27 | -0.14 |
| Household Income | 0.069 | [-0.064, 0.204] | 0.311 | +0 |
| Education (College Degree) Interaction | -0.315 | [-0.582, -0.047] | 0.927 | -0.039 |
| Political Ideology * Education (College Degree) Interaction | -0.09 | [-0.344, 0.163] | 0.485 | -0.064 |
| Intercept | 0.672 | [0.47, 0.876] | 0.082 | 1.17 |
| Model statistics: *n* = 1,124; McFadden’s pseudo-R^2^ = 0.03.  Survey question: “Do you think the government should put less, the same amount, or more effort into: reducing solid waste and garbage?”  Response coding: 1 = *more government effort,* 0 = *the same amount* or *less government effort.* | | | | |
