# Supplementary material for: Younger Americans are less politically polarized than older Americans about climate policies (but not about other policy domains)
Source: PLoS One. 2024 May 15;19(5):e0302434. doi: 10.1371/journal.pone.0302434 (PMC11095675; doi:10.1371/journal.pone.0302434)
Supplement: S27 Table — (DOCX) [file pone.0302434.s031.docx]

**S27 Table. Regression model for addressing global warming survey question (ANES 1996; logistic regression).**

| Variable | Standardized Coefficient (Cohen’s *d*) | Standardized 95% Confidence Interval | *p*-value | Unstandardized Coefficient |
| --- | --- | --- | --- | --- |
| Political Ideology | -0.341 | [-0.52, -0.166] | 0.468 | -0.11 |
| Age | -0.127 | [-0.258, 0.002] | 0.728 | 0.004 |
| Political Ideology * Age Interaction | -0.066 | [-0.203, 0.068] | 0.337 | -0.003 |
| Gender (Male) | 0.001 | [-0.256, 0.259] | 0.993 | 0.001 |
| Household Income | 0.007 | [-0.131, 0.144] | 0.92 | +0 |
| Education (College Degree) Interaction | -0.221 | [-0.501, 0.056] | 0.223 | 0.507 |
| Political Ideology * Education (College Degree) Interaction | -0.24 | [-0.504, 0.022] | 0.073 | -0.169 |
| Intercept | -0.423 | [-0.628, -0.219] | 0.547 | 0.394 |
| Model statistics: *n* = 1,082; McFadden’s pseudo-R^2^ = 0.04.  Survey question: “Do you think the government should put less, the same amount, or more effort into: addressing global warming?”  Response coding: 1 = *more government effort,* 0 = *the same amount* or *less government effort.* | | | | |
