# Supplementary material for: Younger Americans are less politically polarized than older Americans about climate policies (but not about other policy domains)
Source: PLoS One. 2024 May 15;19(5):e0302434. doi: 10.1371/journal.pone.0302434 (PMC11095675; doi:10.1371/journal.pone.0302434)
Supplement: S28 Table — (DOCX) [file pone.0302434.s032.docx]

**S28 Table. Regression model for fuel standards survey question (ANES 2008; linear regression).**

| Variable | Standardized Coefficient (Cohen’s *d*) | Standardized 95% Confidence Interval | *p*-value | Unstandardized Coefficient |
| --- | --- | --- | --- | --- |
| Political Ideology | -0.125 | [-0.222, -0.029] | 0.316 | -0.163 |
| Age | -0.008 | [-0.079, 0.063] | 0.965 | -0.001 |
| Political Ideology * Age Interaction | -0.001 | [-0.073, 0.07] | 0.969 | -0 |
| Gender (Male) | 0.122 | [-0.02, 0.264] | 0.093 | 0.25 |
| Household Income | -0.014 | [-0.091, 0.062] | 0.712 | -0 |
| Education (College Degree) Interaction | 0.084 | [-0.071, 0.24] | 0.019 | 1.015 |
| Political Ideology * Education (College Degree) Interaction | -0.152 | [-0.295, -0.009] | 0.037 | -0.203 |
| Intercept | -0.094 | [-0.212, 0.023] | < 0.001 | 3.107 |
| Model statistics: *n* = 742; multiple R^2^ = 0.05.  Survey question: “Do you favor, oppose, or neither favor nor oppose the federal government requiring automakers to build cars that use less gasoline?” If *favor* or *oppose*, “Do you [favor / oppose] that a great deal, moderately, or a little?”  Response coding: Ranges from 1 = *oppose fuel standards a great deal* to 7 = *favor fuel standards a great deal.* | | | | |
