# Supplementary material for: Younger Americans are less politically polarized than older Americans about climate policies (but not about other policy domains)
Source: PLoS One. 2024 May 15;19(5):e0302434. doi: 10.1371/journal.pone.0302434 (PMC11095675; doi:10.1371/journal.pone.0302434)
Supplement: S29 Table — (DOCX) [file pone.0302434.s033.docx]

**S29 Table. Regression model for power plant emission standards survey question (ANES 2008; linear regression).**

| Variable | Standardized Coefficient (Cohen’s *d*) | Standardized 95% Confidence Interval | *p*-value | Unstandardized Coefficient |
| --- | --- | --- | --- | --- |
| Political Ideology | -0.071 | [-0.168, 0.025] | 0.043 | -0.319 |
| Age | -0.024 | [-0.095, 0.048] | 0.073 | -0.023 |
| Political Ideology * Age Interaction | 0.06 | [-0.011, 0.132] | 0.099 | 0.005 |
| Gender (Male) | 0.004 | [-0.139, 0.147] | 0.955 | 0.008 |
| Household Income | 0.01 | [-0.067, 0.088] | 0.794 | +0 |
| Education (College Degree) Interaction | 0.158 | [0.002, 0.314] | 0.003 | 1.271 |
| Political Ideology * Education (College Degree) Interaction | -0.178 | [-0.321, -0.034] | 0.016 | -0.232 |
| Intercept | -0.077 | [-0.196, 0.041] | < 0.001 | 6.626 |
| Model statistics: *n* = 738; multiple R^2^ = 0.04.  Survey question: “Power plants put gases into the air that could cause global warming. Do you favor, oppose, or neither favor nor oppose the federal government lowering the amount of these gases that power plants are allowed to put into the air?” If *favor* or *oppose*, “Do you [favor / oppose] that a great deal, moderately, or a little?”  Response coding: Ranges from 1 = *oppose emissions regulations a great deal* to 7 = *favor emissions regulations a great deal.* | | | | |
