# Supplementary material for: Younger Americans are less politically polarized than older Americans about climate policies (but not about other policy domains)
Source: PLoS One. 2024 May 15;19(5):e0302434. doi: 10.1371/journal.pone.0302434 (PMC11095675; doi:10.1371/journal.pone.0302434)
Supplement: S30 Table — (DOCX) [file pone.0302434.s034.docx]

**S30 Table. Regression model for gasoline tax survey question (ANES 2008; linear regression).**

| Variable | Standardized Coefficient (Cohen’s *d*) | Standardized 95% Confidence Interval | *p*-value | Unstandardized Coefficient |
| --- | --- | --- | --- | --- |
| Political Ideology | -0.064 | [-0.16, 0.032] | 0.225 | -0.146 |
| Age | 0.103 | [0.033, 0.174] | 0.803 | 0.002 |
| Political Ideology * Age Interaction | 0.029 | [-0.042, 0.099] | 0.429 | 0.002 |
| Gender (Male) | -0.023 | [-0.164, 0.119] | 0.751 | -0.035 |
| Household Income | -0.037 | [-0.113, 0.04] | 0.344 | -0 |
| Education (College Degree) Interaction | -0.119 | [-0.274, 0.036] | 0.014 | 0.781 |
| Political Ideology * Education (College Degree) Interaction | -0.234 | [-0.376, -0.092] | 0.001 | -0.233 |
| Intercept | 0.051 | [-0.066, 0.168] | < 0.001 | 6.512 |
| Model statistics: *n* = 745; multiple R^2^ = 0.06.  Survey question: “Do you favor, oppose, or neither favor nor oppose increasing taxes on gasoline so people either drive less or buy cars that use less gas?” If *favor* or *oppose*, “Do you [favor / oppose] that a great deal, moderately, or a little?”  Response coding: Ranges from 1 = *oppose increasing gasoline taxes a great deal* to 7 = *favor increasing gasoline taxes a great deal.* | | | | |
