# Supplementary material for: Younger Americans are less politically polarized than older Americans about climate policies (but not about other policy domains)
Source: PLoS One. 2024 May 15;19(5):e0302434. doi: 10.1371/journal.pone.0302434 (PMC11095675; doi:10.1371/journal.pone.0302434)
Supplement: S31 Table — (DOCX) [file pone.0302434.s035.docx]

**S31 Table. Regression model for nuclear power plants survey question (ANES 2012; logistic regression).**

| Variable | Standardized Coefficient (Cohen’s *d*) | Standardized 95% Confidence Interval | *p*-value | Unstandardized Coefficient |
| --- | --- | --- | --- | --- |
| Political Ideology | -0.335 | [-0.423, -0.248] | 0.2 | -0.102 |
| Age | -0.227 | [-0.294, -0.161] | 0.612 | -0.003 |
| Political Ideology * Age Interaction | -0.061 | [-0.129, 0.007] | 0.078 | -0.003 |
| Gender (Male) | -1.208 | [-1.341, -1.075] | < 0.001 | -1.208 |
| Household Income | -0.225 | [-0.292, -0.158] | < 0.001 | -0 |
| Education (College Degree) Interaction | -0.405 | [-0.548, -0.261] | 0.424 | -0.172 |
| Political Ideology * Education (College Degree) Interaction | -0.082 | [-0.217, 0.053] | 0.233 | -0.056 |
| Intercept | 1.665 | [1.547, 1.784] | < 0.001 | 3.035 |
| Model statistics: *n* = 4,972; McFadden’s pseudo-R^2^ = 0.11.  Survey question: “Do you think the United States should have more nuclear power plants, fewer nuclear power plants, or the same number it has now?”  Response coding: 1 = *fewer nuclear power plants,* 0 = *more* or *the same number of plants.* | | | | |
