# Supplementary material for: Younger Americans are less politically polarized than older Americans about climate policies (but not about other policy domains)
Source: PLoS One. 2024 May 15;19(5):e0302434. doi: 10.1371/journal.pone.0302434 (PMC11095675; doi:10.1371/journal.pone.0302434)
Supplement: S32 Table — (DOCX) [file pone.0302434.s036.docx]

**S32 Table. Regression model for offshore drilling survey question (ANES 2012; logistic regression).**

| Variable | Standardized Coefficient (Cohen’s *d*) | Standardized 95% Confidence Interval | *p*-value | Unstandardized Coefficient |
| --- | --- | --- | --- | --- |
| Political Ideology | -0.566 | [-0.646, -0.486] | 0.085 | -0.127 |
| Age | -0.274 | [-0.336, -0.213] | 0.419 | 0.005 |
| Political Ideology * Age Interaction | **-0.125** | **[-0.191, -0.058]** | **< 0.001** | -0.005 |
| Gender (Male) | -0.552 | [-0.674, -0.431] | < 0.001 | -0.552 |
| Household Income | -0.164 | [-0.231, -0.097] | < 0.001 | -0 |
| Education (College Degree) Interaction | 0.109 | [-0.032, 0.25] | < 0.001 | 1.43 |
| Political Ideology * Education (College Degree) Interaction | -0.465 | [-0.608, -0.324] | < 0.001 | -0.316 |
| Intercept | 0.051 | [-0.044, 0.146] | < 0.001 | 1.599 |
| Model statistics: *n* = 5,011; McFadden’s pseudo-R^2^ = 0.12.  Survey question: “Offshore Drilling: “Do you favor, oppose, or neither favor nor oppose increased offshore drilling for oil and natural gas in U.S. waters?”  Response coding: 1 = *oppose more offshore drilling,* 0 = *favor more offshore drilling* or *neither.* | | | | |
