# Supplementary material for: Younger Americans are less politically polarized than older Americans about climate policies (but not about other policy domains)
Source: PLoS One. 2024 May 15;19(5):e0302434. doi: 10.1371/journal.pone.0302434 (PMC11095675; doi:10.1371/journal.pone.0302434)
Supplement: S33 Table — (DOCX) [file pone.0302434.s037.docx]

**S33 Table. Regression model for fracking survey question (ANES 2016; logistic regression).**

| Variable | Standardized Coefficient (Cohen’s *d*) | Standardized 95% Confidence Interval | *p*-value | Unstandardized Coefficient |
| --- | --- | --- | --- | --- |
| Political Ideology | 0.775 | [0.6, 0.957] | 0.024 | 0.265 |
| Age | 0.102 | [-0.008, 0.212] | 0.231 | -0.012 |
| Political Ideology * Age Interaction | **0.123** | **[0.011, 0.234]** | **0.03** | 0.004 |
| Gender (Male) | 0.418 | [0.228, 0.608] | < 0.001 | 0.418 |
| Household Income | 0.166 | [0.069, 0.263] | 0.001 | +0 |
| Education (College Degree) Interaction | 0.056 | [-0.172, 0.286] | 0.11 | -0.596 |
| Political Ideology * Education (College Degree) Interaction | 0.251 | [0.023, 0.477] | 0.03 | 0.157 |
| Intercept | -1.842 | [-2.052, -1.641] | < 0.001 | -3.445 |
| Model statistics: *n* = 3,064; McFadden’s pseudo-R^2^ = 0.14.  Survey question: “’Fracking’ is a way to drill for natural gas by pumping high pressure fluid into the ground. Do you favor, oppose, or neither favor nor oppose fracking in the U.S.?”  Response coding: 1 = *oppose fracking,* 0 = *favor fracking* or *neither.* | | | | |
