# Supplementary material for: Younger Americans are less politically polarized than older Americans about climate policies (but not about other policy domains)
Source: PLoS One. 2024 May 15;19(5):e0302434. doi: 10.1371/journal.pone.0302434 (PMC11095675; doi:10.1371/journal.pone.0302434)
Supplement: S34 Table — (DOCX) [file pone.0302434.s038.docx]

**S34 Table. Regression model for federal action on rising temperatures survey question (ANES 2016; linear regression).**

| Variable | Standardized Coefficient (Cohen’s *d*) | Standardized 95% Confidence Interval | *p*-value | Unstandardized Coefficient |
| --- | --- | --- | --- | --- |
| Political Ideology | -0.519 | [-0.569, -0.47] | < 0.001 | -0.335 |
| Age | -0.032 | [-0.062, -0.003] | < 0.001 | 0.021 |
| Political Ideology * Age Interaction | **-0.085** | **[-0.114, -0.056]** | **< 0.001** | -0.006 |
| Gender (Male) | -0.051 | [-0.11, 0.008] | 0.088 | -0.099 |
| Household Income | -0.005 | [-0.036, 0.026] | 0.741 | -0 |
| Education (College Degree) Interaction | -0.003 | [-0.065, 0.06] | 0.102 | 0.282 |
| Political Ideology * Education (College Degree) Interaction | -0.057 | [-0.118, 0.004] | 0.067 | -0.069 |
| Intercept | 0.038 | [-0.018, 0.094] | < 0.001 | 6.496 |
| Model statistics: *n* = 3,046; multiple R^2^ = 0.33.  Survey question: “Do you think the federal government should be doing more about rising temperatures, should be doing less, or is it currently doing the right amount?” If *more* or *less*, “Should it be doing a great deal [more/less], a moderate amount [more/less], or a little [more/less]?”  Response coding: Ranges from 1 = *should be doing a great deal less* to 7 = *should be doing a great deal more.* | | | | |
