# Supplementary material for: Younger Americans are less politically polarized than older Americans about climate policies (but not about other policy domains)
Source: PLoS One. 2024 May 15;19(5):e0302434. doi: 10.1371/journal.pone.0302434 (PMC11095675; doi:10.1371/journal.pone.0302434)
Supplement: S35 Table — (DOCX) [file pone.0302434.s039.docx]

**S35 Table: Annual regression models for federal spending on defense ANES time-series (linear regressions).**

| ANES Year | Standardized Political Ideology * Age Interaction Coefficient (Cohen’s *d*) | Standardized 95% Confidence Interval | *p*-value | Sample Size | Multiple R^2^ |
| --- | --- | --- | --- | --- | --- |
| 1982 | -0.054 | [-0.121, 0.014] | 0.12 | 755 | 0.16 |
| 1984 | -0.049 | [-0.101, 0.003] | 0.07 | 1360 | 0.07 |
| 1986 | 0.017 | [-0.054, 0.088] | 0.64 | 777 | 0.1 |
| 1988 | -0.031 | [-0.084, 0.022] | 0.25 | 1218 | 0.11 |
| 1990 | 0.054 | [-0.004, 0.111] | 0.07 | 1154 | 0.1 |
| 1992 | 0.062 | [-0.006, 0.131] | 0.08 | 846 | 0.1 |
| 1994 | **-0.082** | **[-0.138, -0.025]** | **0.01** | 1230 | 0.13 |
| 1996 | -0.009 | [-0.068, 0.05] | 0.77 | 1043 | 0.14 |
| 1998 | NA |  |  |  |  |
| 2000 | -0.006 | [-0.081, 0.069] | 0.87 | 710 | 0.11 |
| 2002 | 0.056 | [-0.023, 0.136] | 0.17 | 527 | 0.17 |
| 2004 | -0.04 | [-0.104, 0.025] | 0.23 | 770 | 0.17 |
| 2008 | **-0.078** | **[-0.15, -0.007]** | **0.03** | 678 | 0.14 |
| 2012 | 0.027 | [0, 0.054] | 0.06 | 4603 | 0.14 |
| 2016 | 0.019 | [-0.013, 0.05] | 0.24 | 2843 | 0.27 |
| 2020 | **-0.056** | **[-0.077, -0.035]** | **< 0.001** | 5933 | 0.32 |
| Question wording: “Some people believe that we should spend much less money for defense. (Suppose these people are at one end of a scale, at point 1.) Others feel that defense spending should be greatly increased. (Suppose these people are at the other end, at point 7.) And, of course, some other people have opinions somewhere in between, at points 2, 3, 4, 5 or 6. Where would you place yourself on this scale, or haven’t you thought much about this?” This survey question was not asked in 1998.  Response coding: Ranges from 1 = *greatly decrease defense spending* to 7 = *greatly increase defense spending.*  Models controlled for political ideology, age, education, the interaction between education and political ideology, gender and household income. | | | | | |
