# Supplementary material for: Younger Americans are less politically polarized than older Americans about climate policies (but not about other policy domains)
Source: PLoS One. 2024 May 15;19(5):e0302434. doi: 10.1371/journal.pone.0302434 (PMC11095675; doi:10.1371/journal.pone.0302434)
Supplement: S36 Table — (DOCX) [file pone.0302434.s040.docx]

**S36 Table: Annual regression models for public versus private health insurance ANES time-series (linear regressions).**

| ANES Year | Standardized Political Ideology * Age Interaction Coefficient (Cohen’s *d*) | Standardized 95% Confidence Interval | *p*-value | Sample Size | Multiple R^2^ |
| --- | --- | --- | --- | --- | --- |
| 1982 | NA |  |  |  |  |
| 1984 | -0.063 | [-0.141, 0.016] | 0.12 | 627 | 0.06 |
| 1986 | NA |  |  |  |  |
| 1988 | -0.004 | [-0.058, 0.051] | 0.9 | 1162 | 0.1 |
| 1990 | NA |  |  |  |  |
| 1992 | -0.02 | [-0.091, 0.051] | 0.59 | 793 | 0.09 |
| 1994 | -0.034 | [-0.09, 0.021] | 0.22 | 1230 | 0.19 |
| 1996 | 0.015 | [-0.041, 0.071] | 0.61 | 1057 | 0.2 |
| 1998 | NA |  |  |  |  |
| 2000 | 0.019 | [-0.053, 0.091] | 0.61 | 778 | 0.08 |
| 2002 | NA |  |  |  |  |
| 2004 | 0.022 | [-0.04, 0.083] | 0.49 | 785 | 0.22 |
| 2008 | **-0.072** | **[-0.141, -0.003]** | **0.04** | 697 | 0.18 |
| 2012 | **-0.032** | **[-0.056, -0.007]** | **0.01** | 4757 | 0.27 |
| 2016 | -0.011 | [-0.041, 0.019] | 0.48 | 2860 | 0.33 |
| 2020 | 0.001 | [-0.018, 0.02] | 0.92 | 6054 | 0.42 |
| Question wording: “There is much concern about the rapid rise in medical and hospital costs. Some people feel there should be a government insurance plan which would cover all medical and hospital expenses for everyone. Suppose these people are at one end of a scale, at point 1. Others feel that all medical expenses should be paid by individuals through private insurance plans like Blue Cross or other company paid plans. Suppose these people are at the other end, at point 7. And, of course, some other people have opinions somewhere in between, at points 2, 3, 4, 5, or 6. Where would you place yourself on this scale, or haven’t you thought much about this?” This survey question was not asked in 1982, 1986, 1990, 1998, and 2002.  Response coding: Responses were reversed-scored such that they ranged from 1 = *private insurance plan* to 7 = *government insurance plan*.  Models controlled for political ideology, age, education, the interaction between education and political ideology, gender and household income. | | | | | |
