# Supplementary material for: Younger Americans are less politically polarized than older Americans about climate policies (but not about other policy domains)
Source: PLoS One. 2024 May 15;19(5):e0302434. doi: 10.1371/journal.pone.0302434 (PMC11095675; doi:10.1371/journal.pone.0302434)
Supplement: S37 Table — (DOCX) [file pone.0302434.s041.docx]

**S37 Table: Annual regression models for federal spending on welfare ANES time-series (logistic regressions).**

| ANES Year | Standardized Political Ideology * Age Interaction Coefficient (Cohen’s *d*) | Standardized 95% Confidence Interval | *p*-value | Sample Size | McFadden's pseudo-R^2^ |
| --- | --- | --- | --- | --- | --- |
| 1982 | **-0.236** | **[-0.448, -0.03]** | **0.03** | 783 | 0.09 |
| 1984 | NA |  |  |  |  |
| 1986 | NA |  |  |  |  |
| 1988 | NA |  |  |  |  |
| 1990 | NA |  |  |  |  |
| 1992 | -0.091 | [-0.293, 0.108] | 0.37 | 854 | 0.06 |
| 1994 | -0.125 | [-0.31, 0.056] | 0.18 | 1249 | 0.08 |
| 1996 | -0.104 | [-0.328, 0.116] | 0.36 | 1128 | 0.11 |
| 1998 | NA |  |  |  |  |
| 2000 | -0.155 | [-0.312, -0.001] | 0.05 | 1415 | 0.07 |
| 2002 | -0.162 | [-0.38, 0.052] | 0.14 | 582 | 0.08 |
| 2004 | 0.09 | [-0.082, 0.261] | 0.3 | 819 | 0.09 |
| 2008 | -0.035 | [-0.152, 0.082] | 0.56 | 1470 | 0.06 |
| 2012 | **-0.125** | **[-0.211, -0.039]** | **< 0.001** | 5032 | 0.12 |
| 2016 | -0.025 | [-0.138, 0.087] | 0.66 | 3078 | 0.17 |
| 2020 | 0.024 | [-0.051, 0.098] | 0.53 | 6505 | 0.22 |
| Question wording: “Should federal spending on welfare programs be increased, decreased, or kept the same?” Slightly different question wording was used in 1982; please refer to question wording and coding decisions section. This survey question was not asked in 1984, 1986, 1988, 1990, or 1998.  Response coding: 1 = *increased*, 0 = *decreased* or *kept the same.*  Models controlled for political ideology, age, education, the interaction between education and political ideology, gender and household income. | | | | | |
