# Supplementary material for: Younger Americans are less politically polarized than older Americans about climate policies (but not about other policy domains)
Source: PLoS One. 2024 May 15;19(5):e0302434. doi: 10.1371/journal.pone.0302434 (PMC11095675; doi:10.1371/journal.pone.0302434)
Supplement: Attachment — Submitted filename: Response to Reviewers.docx [file pone.0302434.s042.docx]

Letter to Editor Addressing Reviewer Feedback

Hi Jose & PLOS ONE editors,

Thank you for sharing the thoughtful reviews of our manuscript “Younger Americans are less politically polarized than older Americans about climate policies (but not about other policy domains)”, which we submitted to PLOS ONE.

As you will see in our comments below and in the revised manuscript, we have carefully incorporated the suggestions from Reviewer 1. As requested, we have improved the clarity of our paper by adding to our literature review to better ground our analyses with current research in polarization and climate policy, and by re-organizing content across certain sections to enhance flow. We hope that you agree that the current revision is now suitable for publication in PLOS ONE.

Sincerely,

Joshua Inwald and co-authors

Reviewer 1 Feedback

**Introduction**

I find the introduction somewhat limited and burdened with references that could or should be reduced, as they do not contribute additional information and relevance to the argument. For instance, references 8, 9, and 10 pertain to three documents on the same 2019 survey (World Risk Poll), and references 14, 15, and 16, two of which are studies from the Yale Program on Climate Change, widely known and referencing one of them would likely suffice.

AUTHOR RESPONSE: We have reduced the number of citations to these datasets, and now only cite one paper for each dataset (World Risk Poll, Yale Program on Climate Change) that is most relevant to the literature review argumentation.

Furthermore, these references are not utilized to compare and discuss the results in the discussion section. However, new references are introduced in the discussion section to analyze the findings. In my experience in reading, writing, or reviewing articles, as well as in various peer review training courses and seminars I have attended, it is customary for the introduction and the state of the art to present the literature guiding and contextualizing the study. Subsequently, the results are discussed in relation to the previously mentioned literature. This structure is not evident in this work. Out of the 27 citations in this introductory section, only 2 (citations 21 and 23) are used to discuss the study's results.

AUTHOR RESPONSE: To improve the consistency of the paper, we now discuss possible mechanisms for differential polarization based on age in the Introduction section (lines 112 – 134), as well as returning to these same arguments and sources in the discussion (lines 458 – 481).

I believe there is a need for a more in-depth literature review that positions the reader in the current state of research, particularly regarding ideological polarization among different age groups concerning climate policies and other political dimensions unrelated to climate change. To achieve this, you can make use of references 39 to 48, which you have employed to discuss your results. Additionally, I recommend exploring studies by Professor Dan Kahan of the Cultural Cognition Project (http://culturalcognition.squarespace.com/), where you will find research on political polarization, worldviews, and perceptions on climate and other controversial topics (weapons, nuclear energy, etc.).

AUTHOR RESPONSE: We have added to the literature review on Americans’ polarization of climate change policies, including perspectives from the Cultural Cognition literature and a competing theoretical camp that argues climate polarization stems from scientific literacy and motivated reasoning (lines 71 – 91). We also mention how these theories might also apply to understanding of polarization of other policy topics.

**Materials and Methods**

I suggest restructuring this section to enhance clarity and provide a transparent presentation of the followed methodological process.

The section begins by describing the work of the American National Election Studies (ANES), which is logical and coherent as the source of secondary data for the study. However, it would be beneficial to include information on where these data can be accessed. The current description is limited to a 6-line paragraph, and subsequent sub-sections continue to provide details about ANES that I believe should be incorporated at the beginning of this section, such as the paragraph between lines 127 and 130.

AUTHOR RESPONSE: We now mention ANES access information at the start of the methods section (line 166). We also have moved the ANES details formerly at lines 127 130 to earlier in the Methods section, to improve clarity (see lines 168 – 177).

Moreover, in my opinion, the information provided in Table 1 may not be highly relevant and could be included in supplementary materials. The footnote information could be incorporated into the description of the ANES surveys at the start of the section. If deemed necessary by the authors, they can briefly refer to the relevant information from the table. Additionally, it would be useful to include a sub-section (2.1) with a title for this ANES description.

AUTHOR RESPONSE: The old Table 1 has been moved to Supporting Information (now S1 Table), and we now mention relevant information from the old footnote in the new table of main variables of interest (the new Table 1, see reviewer’s next comment). We also now sub-section the Methods section differently (new sub-sections: 1 ANES overview, 2 ANES participants, 3 ANES survey questions, 4 dependent variable coding, 5 Analytical Strategy), as suggested, to improve clarity.

In sub-section 2.2, Measures, I would take the opportunity to include a table describing the variables of interest, given the removal of Table 1. This suggestion aims to allow readers to easily refer back and identify the variables discussed throughout the article. For example:

Variable Wording Scale
Independent variables Liberal-Conservative Political Ideology Where would you place yourself on this scale, or haven’t you thought much about this? 7-point scale…
Age Birth year, month and day
Climate policy time-series items Federal Spending on the environment
Environment regulations…
Non-climate time-series items Federal Spending…
Private versus…
Federal Spending on…

AUTHOR RESPONSE: We now include a single table summarizing the main variables (political ideology, age, climate policy time series items, non-climate policy time series items) used in the study and their survey question wording and response options. This table is now Table 1 in the manuscript (line 192), appearing at the end of the Methods sub-section 2.3. The old Table 2, which discussed all non-time series climate policy items, has been moved to Supporting Information, Table S2.

Regarding Table 2, which displays other questions about climate policy, I don't believe many of these items are directly related to climate policies; rather, they pertain more to environmental policies. In my opinion, the first 9 items do not align with climate policies but instead focus on environmental, well-being, and social health considerations. It is from the item "Address Global warming" to "Regulations on GHG emitters" that the questions become directly associated with climate policies.

AUTHOR RESPONSE: We now explicitly mention that ANES asks questions about the environment and climate separately, but for brevity, we refer to these items collectively as “climate policy items” (line 42 & lines 197 – 204). We have also moved Table 2 to Supporting Information and re-named it to “ANES Environment and Climate Policy Questions and Response Scales” to reflect the variety of survey question topics, see Table S2 in Supporting Information.

Finally, I fail to see the relevance of reference (31). I suggest considering the possibility of removing it.

AUTHOR RESPONSE: We have removed Reference #31.

**Results**

In this section, I also have some questions. Shouldn't the strategic analysis be included in the methods section? This may be a matter of personal opinion or preferences, but upon reaching this section, I expected to start reading about results. However, the explanation of methodological aspects of the study continues. Don't you think this breaks the flow? Moreover, you previously explained the variables of interest in the study. Nevertheless, in this section, you revisit explanations about these variables and others like educational level, which are ultimately not included in the model. I believe the content of this section up to sub-section 3.2 should be incorporated into the methods section, please consider if it's appropriate.

AUTHOR RESPONSE: We have moved the analytical strategy discussion to the Methods section, to improve the flow of the manuscript, and now the Results section immediately begins by answering Research Question 1 (line 295).

Additionally, I think the R programming language and its various packages are widely known and may not require references for support and justification (references 36 and 37).

AUTHOR RESPONSE: We include references to the R packages used in accordance with PLOS One submission guidelines — <https://journals.plos.org/plosone/s/submission-guidelines#:~:text=List%20the%20name%20and%20version,used%2C%20alongside%20any%20relevant%20references>

Furthermore, consider whether it would be interesting to present a diagram where readers can observe the regression models and how variables respond to these models.

AUTHOR RESPONSE: We now include a visualization of the main regression model at the end of the Methods section, right after outlining the analytical strategy (see Fig 1, line 276). This model highlights the main relationships of interest (IVs: interaction between political ideology and age, political ideology and age; DVs: policy support items; plus modeling controls).

Regarding the rest of the section, as I mentioned earlier, I find it a bit overwhelming due to the number of variables used. Having the suggested table, which systematically describes the variables used, might make reading the results easier. In any case, I encourage you to attempt restructuring this section so that you are clearly answering the research questions. I believe this could enhance the understanding of this section and its results.

AUTHOR RESPONSE: We have addressed this by 1) including the suggested table summarizing main variables in the Methods section, 2) moving details on non-main variables to Supporting Information, 3) including a visual representation of our analytical methods, and 4) re-organizing content between methods vs results to streamline presentation of key findings.

**Discussion**

As I mentioned earlier, I believe that for this discussion section to have coherence and meaning, it would be necessary to establish a prior state of the art, anticipating the reader's understanding of the study's context and allowing the conclusion of the study with this final discussion section.

AUTHOR RESPONSE: We have enhanced the coherence of the Discussion section by 1) improving alignment in topics and references across Introduction and Discussion, and 2) featuring more in-depth discussion of state-of-the-art in research ideas relating to theories of political polarization and how that might vary with Americans’ age in the climate domain, see lines 449 – 481.
